# Promoting better mental health care for patients with psychosis by focusing on differences in causal beliefs between patients and clinicians

**DOI:** 10.1192/j.eurpsy.2022.2010

**Published:** 2022-09-01

**Authors:** R. Rosenthal Oren, D. Roe, I. Hasson-Ohayon, Y. Zisman-Ilani

**Affiliations:** 1 University of Haifa, Department Of Community Mental Health, Haifa, Israel; 2 Bar Ilan University, Department Of Psychology, Ramat Gan, Israel; 3 Temple University, Department Of Social And Behavioral Sciences, Philadelphia, United States of America

**Keywords:** causal beliefs, Psychosis

## Abstract

**Introduction:**

Nonadherence to antipsychotic medications and disengagement from psychiatric services are frequent among people with psychosis. Research indicates how the beliefs of people with psychosis about the etiology of their symptoms, or their causal beliefs, affect treatment choice and outcomes. Yet, there is less research on causal beliefs of clinicians or on the impact of patient–clinician disagreements on treatment and adherence.

**Objectives:**

This review aimed to explore the scope of the literature focusing on clinicians’ causal beliefs and to map the degree of patient–clinician concordance in causal beliefs.

**Methods:**

A systematic literature search of PubMed, Embase, Scopus, PsycInfo, and ASSIA and a grey literature search of PsyArXiv and MedNar yielded 11,821 eligible references.

**Results:**

Forty-two articles indicated that whereas clinicians endorse mainly biogenetic beliefs (9/15 articles, 60%), patients endorse mainly psychosocial causal beliefs (16/31, 52%) and other non-biogenetic causal beliefs (8/31, 26%). Most studies did not compare causal beliefs of people with psychosis and their treating clinicians.

**Conclusions:**

While clinicians and people with psychosis often hold complex causal models, a gap in causal beliefs between these groups appears to exist, which may affect the therapeutic relationship and pose barriers to treatment adherence. Future studies should address this gap by developing interventions that facilitate open communication about causal beliefs to promote treatment alliance and an agreed-on treatment plan.

**Disclosure:**

No significant relationships.

